# Groundnut production constraints and farmers’ trait preferences: a pre-breeding study in Togo

**DOI:** 10.1186/s13002-018-0275-y

**Published:** 2018-11-29

**Authors:** Essohouna Modom Banla, Daniel Kwadjo Dzidzienyo, Ifie Elohor Beatrice, Samuel Kwame Offei, Pangirayi Tongoona, Haile Desmae

**Affiliations:** 1 Togolese Research Institute of Agriculture (ITRA), 13BP267 Lome, Togo; 2 West Africa Centre for Crop Improvement (WACCI), University of Ghana (UG), PMB 30, Legon, Accra, Ghana; 3 International Crops Research Institute for the Semi-Arid tropic (ICRISAT-WCA), BP320 Bamako, Mali

**Keywords:** Groundnut, Participatory rural appraisal, Survey, Breeding, Varieties, Late leaf spot

## Abstract

**Background:**

Groundnut is an important legume crop in Togo. However, groundnut yield has been steadily decreasing for decades as a result of lack of organized breeding program to address production constraints. Though, low yielding varieties and late leaf spot have been often reported as the most important constraints, there is no documented evidence. Identifying and documenting the major production constraints is a prerequisite for establishing a good breeding program with clearly defined priority objectives and breeding strategies. Thus, the objectives of this study were to identify groundnut production constraints and assess farmers’ preferred traits.

**Methods:**

A participatory rural appraisal approach was used to collect data on agronomic practices, farmers’ preferences, and possible threats to production through individual and group interviews. Three regions and three villages per region were selected based on the representativeness of groundnut production systems. In each village, 20 farmers were randomly selected and interviewed; thus, a total of 180 farmers were interviewed. Content analysis was carried out for qualitative data and for quantitative data generated within and across regions, comparative descriptive statistics were carried out. Differences in perception and preferences were assessed using chi-square tests.

**Results:**

The study has revealed that, though there were some variation across the regions, traits pertaining to yield such as pod yield (66.66%) and pod size (12.12%) were the most important. Leaf spot diseases, rosette and peanut bud necrosis (37.77%) and insects such as pod sucking bug and bruchid (27.77%) were considered to be the most important constraints limiting groundnut production. Among diseases, farmers in all the three regions indicated that late leaf spot is of economic importance which they associated to various causes such as maturity, drought, or insects. No gender differences were observed for the perception of constraints and groundnut traits preferences. Land size is significantly influenced by age and gender. Besides, farmers have pointed the lack of improved varieties and the unavailability of groundnut seeds highlighting the necessity of a sustainable groundnut seed system linked with a strong breeding program.

**Conclusion:**

This study has enabled understanding of the farming practices, constraints, and farmers preferred characteristics, thus providing the basis for a participatory breeding program in Togo which should consider that farmers perceive low yielding varieties and diseases as major constraints to production.

## Background

Recently, the awareness on the failure of conventional methods of agricultural projects led to a search for alternative approaches to generate information for development of new technologies [1, 2]. One widely adopted strategy is the participatory rural appraisal (PRA), which is intended to enable local farmers’ participation to conduct their own analysis and to influence the direction of the research [3, 4]. Farmers’ participation guarantees that the new developed technologies will be easily adopted and farmers could play a key role in the diffusion of those technologies [5, 6]. In the case of plant breeding, the direct involvement of farmers as an integral part of the process ensures the participation of the most important stakeholders in the breeding activities [6, 7]. The objective of almost all the participatory breeding approaches has been to incorporate farmers’ knowledge and preferences into the breeding program in order to develop new varieties that will not be rejected by them [8–11]. Results of PRA studies are exploited in the identification of the parents to be crossed to develop new population for selection. Also, PRA results speed the breeding by helping the breeder to focus on the most desired traits [12, 13]. Some studies have noted gender difference in farmers’ preferences in many African countries [14–17]. It is important, therefore, to have in mind the role played by men and women when conducting a survey for the identification of production constraints and farmers’ desired traits [17]. Also, addressing gender differences in implementing agricultural project results in greater impact on farmers’ livelihood [18] as gender affects adoption of new agricultural technologies [13, 19].

In Togo, groundnut and cowpea are the two most important legume crops grown by smallholder farmers for home consumption and market. Unfortunately, groundnut has not gained as much research attention as it deserves. There is no organized breeding program and very few improved varieties available to farmers were old introductions from adaptation tests of varieties released in other countries. As a consequence, groundnut yield has not been increasing, rather steadily decreasing over the years with current yield estimated to be around 0.6 t/ha [20]. Recently, there is a focus shift where establishing an organized breeding program has gained priority to develop improved varieties that boost groundnut productivity and production in Togo. This is led by the national institute of agricultural research (ITRA) with the recruitment and capacity building of a breeder. Efforts have been going on to identify production constraints and understanding farmers’ preference criteria, identify target population of environments, establishing test environments, and assembling gene pools. Though, low yielding varieties and late leaf spot (LLS) has been often reported by agricultural extension agents as the most important constraints limiting groundnut production in Togo, no scientific method has been used to ascertain groundnut production constraints. Therefore, as part of starting the new breeding program, a PRA was conducted in three regions of Northern Togo. This pre-breeding survey, the first study with regards to groundnut production in Togo, is aimed at identifying groundnut production constraints, assessing farmers’ knowledge of late leaf spot disease, and identifying farmers’ preferred traits. The information on production constraints will help rank them according to their importance including agronomic (e.g., variety, cultural practices), abiotic (drought), and biotic (e.g., late leaf spot, insect pests) constraints. Also, production constraints are not often fully independent, rather complex. The assessment of farmers’ knowledge of late leaf spot disease will help to understand farmers perception and management strategies of late leaf spot. The study on farmers’ preferences will help to identify priority traits and provides an insight on gender differences. Therefore, results from the PRA will guide the breeding program in defining priority constraints and traits, and in developing breeding strategies to develop improved varieties that are high yielding, adapted to target environments, resistant to biotic and abiotic constraints, and preferred by farmers, market, and consumers.

## Methodology

### Description of the study area

**Fig. 1:**
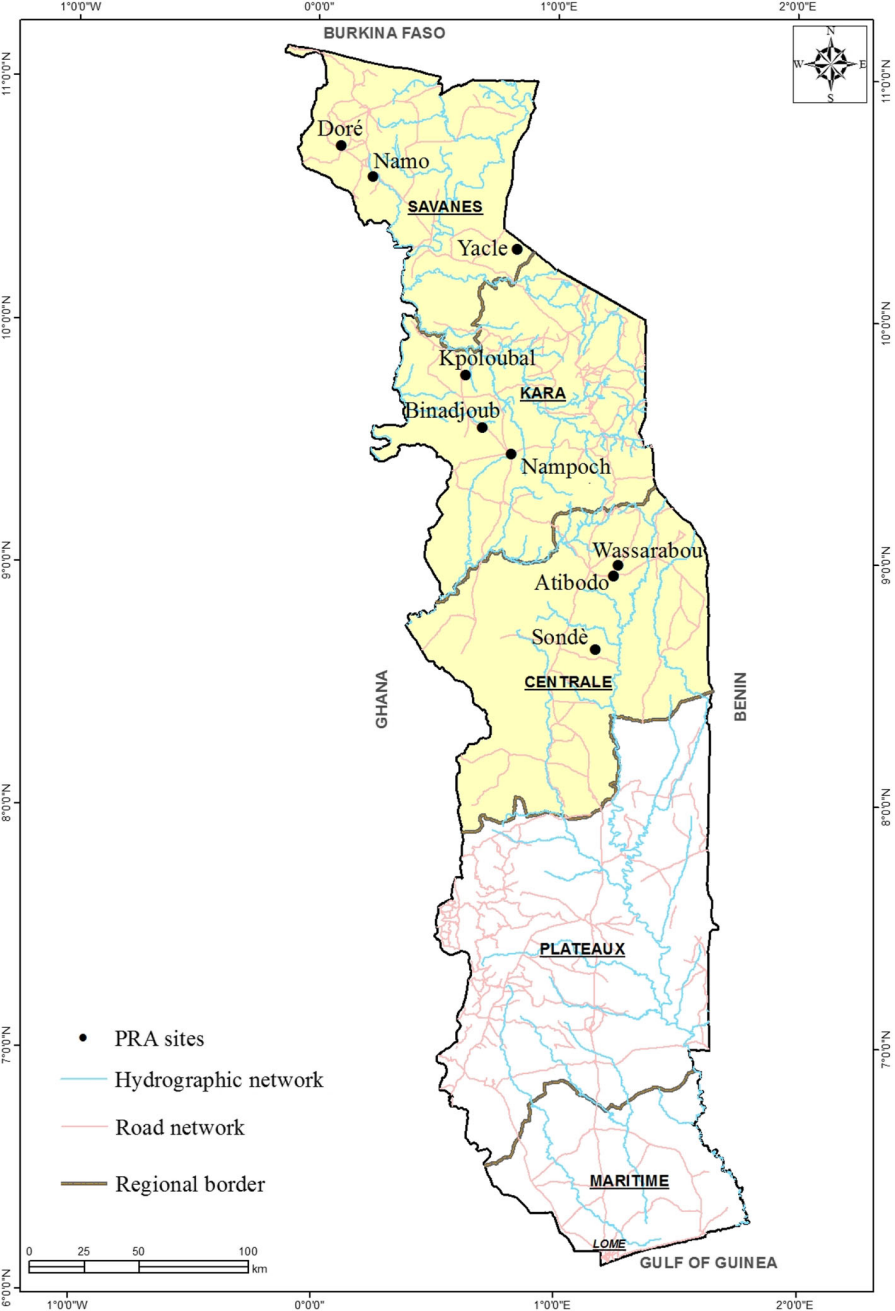
Map of Togo showing the surveyed area (ArcGIS 10.2.2)

The study area comprises three regions: Savanes, Kara, and Centrale located in the north of the country (Table 1; Fig. 1). The three regions share farming and trading as the major socio-economic activity. In addition to the farming and trading, Savanes and Centrale, to a lesser extent, are characterized by livestock keeping. These northern regions are in the Sudano-Sahelian zone experiencing a single wet season from April to October [21, 22]. Three villages per region were selected based on criteria such as importance of groundnut in local farming systems, representativeness of farmers, ecological, and socio-economic conditions.

**Table 1:** Sites and the number of farmers interviewed during the survey

Region	Village	Name of the community	Geographical location	No. of FGD	No. of farmers
Kara	Kpoloubal	Bassar	N 09.77990 E 00.62039	1	20
	Binadjoub	Kabye	N 09.56500 E 00.69254	1	20
	Nampoch	Lamba	N 09.22001 E00.71510	1	20
Savanes	Yacle	Moba	N 10.29908 E 00.79510	1	20
	Dore	Bissa	N 10.72191 E 00.09526	1	20
	Namo	Moba	N 10.33601 E 00.74864	1	20
Centrale	Wassarabou	Kotocoli	N 08.95293 E 01.21332	1	20
	Atibodo	Kabye	N 08.95458 E 01.24128	1	20
	Sonde	Kabye	N 08.562680 E 00.973260	1	20
Maritime	Gboto	Ewe	N 06.67739 E 01.53206	1	0
	Tabligbo	Ewe	N 06.588117 E 01.499870	1	0
Total	12	7		11	180

*No. of FGD number of focus group discussion

### Survey and data analysis

The survey team was guided by some prior knowledge of the distribution and importance of groundnut obtained from the Direction of National Agricultural Statistics [20] and from the agricultural extension officers. Preliminary visits were made to discuss with the extension agents and the farmers. These visits provided opportunities to pre-test the questionnaire and at the same time to collect preliminary data on groundnut production system and constraints. According to the national statistics data [20], the mean number of groundnut farmers per village ranges from 53 to 93 in the surveyed area. Thus, from a list of groundnut farmers provided by the agricultural extension service, 20 farmers were randomly selected in each village resulting in a total number of 180 farmers interviewed in the three northern regions (Table 1).

Demographic information such as household structure, educational level, and land property were obtained for the randomly selected farmers. Table 2 presents the age of groundnut farmers while Table 3 contains information on family size and literacy level. The sex and marital status of the groundnut farmers are summarized in Table 4. Majority of the farmers (62.77%) were 50 years old and below with the most predominant age group of farmers being between 41 and 50 years old. The next largest age groups in rank were farmers between 51 and 60 years (26.11%) and more than 61 years (11.11%). However, at the region level, most of the farmers were aged less than 40 years in Kara (38.33%), between the ages of 41 and 50 years both in Savanes (31.67%) and Centrale (36.67%). Also, Savanes had relatively the largest population of farmers that are more than 61 years old (21.67%).

**Table 2:** Age of groundnut farmers interviewed in Togo

Region	Village	Age							
		< 40		41–50		51–60		> 61	
		Num.*	Perc.	Num.	Perc.	Num.	Perc.	Num.	Perc.
Kara	Kpoloubal	7	35	10	50	2	10	1	5
	Binadjoub	8	40	6	30	4	20	2	10
	Nampoch	8	40	4	20	8	40	0	0
	Total/mean	23	38.33	19	33.33	14	23.34	3	5
Savanes	Yacle	4	20	6	30	6	30	4	20
	Dore	3	15	7	35	5	25	5	25
	Namo	4	20	6	30	6	30	4	20
	Total/mean	11	18.33	19	31.67	17	28.33	13	21.67
Centrale	Sonde	4	20	10	50	6	30	0	0
	Kitambouli	6	30	6	30	6	30	2	10
	Wassarabou	8	40	6	30	4	20	2	10
	Total/mean	18	30	22	36.67	16	26.67	4	6.66
Grand total/mean		52	28.89	61	33.89	47	26.11	20	11.11

*Num. = number; *Perc.* = percentage

**Table 3:** Educational qualification and household size of groundnut farmers in Togo

Region	Village	Qualification										Typical household
		Illiterate		Lit. tuition*		PS		SS		Degree		
		Num.	Perc.	Num.	Perc.	Num.	Perc.	Num.	Perc.	Num.	Perc.	
Kara	Kpoloubal	9	45	6	30	4	20	1	5	0	0	9
	Binadjoub	8	40	2	10	5	25	5	25	0	0	7
	Nampoch	10	50	0	0	8	40	2	10	0	0	6
	Total/mean (%)	27	45	8	13.33	17	28.33	8	13.34	0	0	7.33ab**
Savanes	Yacle	12	60	0	0	2	10	6	30	0	0	6
	Dore	3	15	7	35	6	30	4	20	0	0	11
	Namo	12	60	0	0	2	10	6	30	0	0	6
	Total/mean (%)	27	45	7	11.66	10	16.66	16	26.68	0	0	8.33a
Centrale	Sonde	9	45	4	20	3	15	4	20	0	0	7
	Attibodo	7	35	6	30	3	15	4	20	0	0	6
	Wassarabou	6	30	7	35	4	20	3	15	0	0	6
	Total/mean (%)	22	36.66	17	28.33	10	16.67	11	18.34	0	0	6.33b
Grand total/mean (%)		76	42.22	32	17.78	37	20.55	35	19.45	0	0	7.33

**Lit. tuition = literacy tuition; *PS* = primary school; *SS*=secondary school; *Num.* = number; *Perc.* = percentage

*Means within a column followed by the same letter(s) are not significantly different

**Table 4:** Sex and marital status of groundnut farmers in study area of Togo

Region	Village	Sex				Marital status					
		Male		Female		Married		Single		Widower	
		Num.	Perc.	Num.	Perc.	Num.	Perc.	Num.	Perc.	Num.	Perc.
Kara	Kpoloubal	15	75	5	25	20	100	0	0	0	0
	Binadjoub	13	65	7	35	19	95	1	5	0	0
	Nampoch	4	20	16	80	20	100	0	0	0	0
	Total/mean (%)	32	53.33a*	28	46.67	59	98.33	1	1.67	0	0b
Savanes	Yacle	18	90	2	10	18	90	0	0	2	10
	Dore	12	60	8	40	18	90	2	10	0	0
	Namo	13	65	7	35	15	75	2	10	3	15
	Total/mean (%)	43	71.66b	17	28.33	51	85	4	6.67	5	8.33a
Centrale	Sonde	16	80	4	20	18	90	2	10	0	0
	Attibodo	11	55	9	45	19	95	0	0	1	5
	Wassarabou	10	50	10	50	17	85	3	15	0	0
	Total/mean (%)	37	61.66ab	23	38.33	54	90	5	8.33	1	1.67b
Grand total/mean (%)		112	62.22	68	37.78	164	91.11	10	5.56	6	3.33

*Num. = number; *Perc.* = percentage

**Means within a column followed by the same letter(s) are not significantly different

**Fig. 2:**
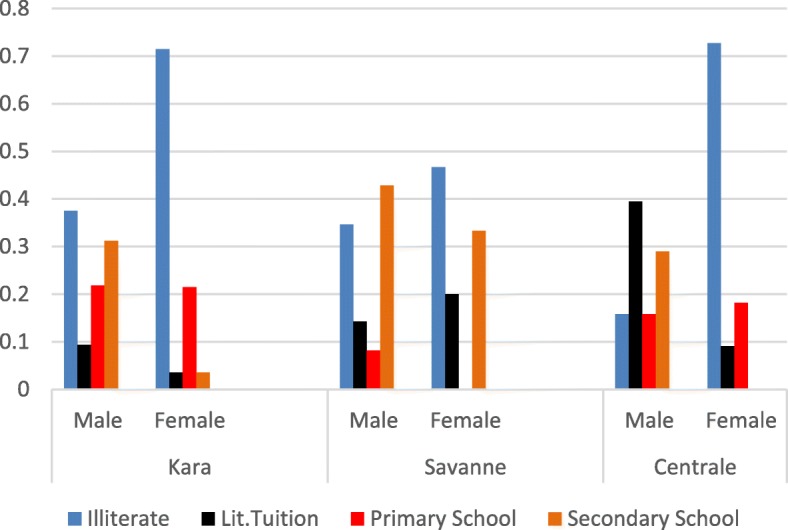
Educational qualification by gender (in percentage of interviewees)

With regards to literacy level, only 20.55% of the farmers own a Primary School Certificate and 19.45% a Secondary School Certificate. Thus, many of the farmers (42.22%) were illiterate. Between the literate group and the illiterate group, 17.77% of the respondents have had some form of literacy tuition. At the region level, Centrale appears relatively more educated with only 36.66% illiteracy (Fig. 2). The average family size is seven in Centrale, eight in Kara, and nine in Savanes.

The sex distribution of the groundnut farmers indicated that more males (62.22%) than females (37.78%) are engaged in groundnut production in the three regions of Togo. Region wise, the number of females is higher in Kara (46.67%) than in the other regions. In Kara, the number of females is higher than males in Nampoch (80%) unlike in Yacle (Savanes) where men represent 90% of the farmers. Most of the groundnut farmers are married (91.11%). The remaining farmers were either unmarried (5.56%) or widowers (3.33%).

In addition to individual interviews, two focal group discussions were carried out in the Maritime (South) region to check the information that farmers are increasingly interested in growing groundnut. Agricultural extension workers and village chiefs were of a great help in this process because of their familiarity with farmers. In each selected village, one villager was identified as local representative for preparing the focus group discussion session on completion of the questionnaire interviews. Through individual and group interviews, information on the cultivated varieties, the agronomic practices, the use of groundnut, and the production constraints were collected. Scoring and ranking were used to access farmers’ constraints and preferences. With a focus on late leaf spot, farmers’ knowledge and perception of the constraint as well as management options were assessed. Gender differences were assessed to ascertain the roles played by both men and women and their constraints in groundnut production as well as preferred traits. Where possible, small sample of groundnut seeds were collected from the farmers.

The “Survey Package” version 3.7 [23] was used for the analysis of quantitative data collected through individual questionnaires in R version 3.3.1. For data collected within and across regions, analysis of variance was carried out for demographic and farm characteristics data. Comparative descriptive statistics were carried and percentage and means were used for the presentation of results related to perception and preferences. Chi-square test was used for the comparison of the perception and preferences between regions and between genders. The association between social and farming system parameters was done through Pearson’s correlation analysis [24]*.* Content analysis was carried out for qualitative data [25] collected at both individual and the group level to gain in-depth understanding of farmers’ perception of constraint and preferences. Using geographic data collected, ArcGIS version 10.2.2 [26] was used to draw the map of Togo showing the PRA sites.

## Results

### Groundnut production practices

Groundnut is cultivated mainly in the rainy season. The land is cleared and plowed in April (in Centrale), May (in Kara), and June (in Savanes) by both men and women but mostly by hired men. Tractors, hoes, axes, and to a lesser extent ox-plows are the main equipment used by farmers. Sowing follows the first rain after plowing. Usually, weeding is done twice by both men and women around June, July, and at late August. Incorporating manure from cattle, goats, and sheep on their crops is not common, as animals graze freely. In addition, farmers reported that there is no use of inorganic fertilizer except in mixed cropping when fertilizers are applied to maize.

### Farm characteristics

**Fig. 3:**
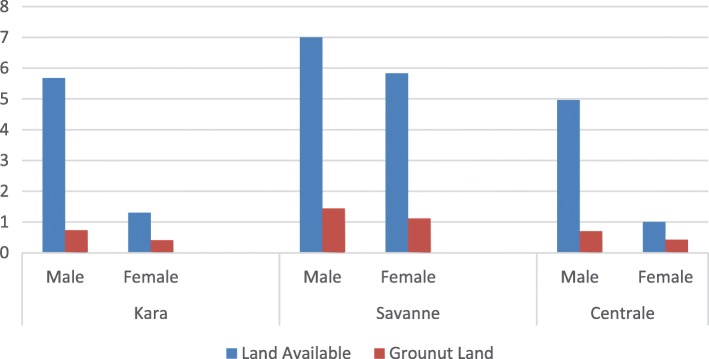
Total area cultivated and part for groundnut (ha) as estimated by interviewees

The average groundnut farm size is 0.58 ha in Kara, 0.6 ha in Centrale, and 1.35 ha in Savanes (Table 5). In general, the average groundnut farm size in the surveyed areas is 0.84 ha, with the smallest being 0.44 ha and the largest being 1.66 ha. Analysis of variance showed that there is a significant difference across regions (*p* < 0.001) and between villages (*p* < 0.01). There is also a clear gender difference in land available for agriculture in all the three regions (Fig. 3). Men have access to larger land than women. As a consequence, women’s groundnut plot is smaller than men’s plot.

**Table 5:** Farm characteristics from the three regions of Togo

Region	Village	Groundnut area range (ha)			Seed Source			% M.C.**	Sold Proportion (%)
		Smallest	Average	Largest	Market	Saved	Others		
Kara	Kpoloubal	0.25	0.51	0.75	30.00	60.00	10.00	60.00	60.00
	Binadjoub	0.25	0.71	2.00	45.00	50.00	5.00	95.00	75.5
	Nampoch	0.25	0.52	0.75	05.00	75.00	20.00	80.00	59.2
	Mean	0.25	0.58b	1.16	26.67	61.67	11.66	78.33	64.90b
Savanes	Yacle	1	1.47	2.00	20.00	65.00	15.00	60.00	93.50
	Dore	0.5	1.12	2.00	25.00	75.00	0.00	7.00	83.75
	Namo	1	1.47	2.00	30.00	70.00	0.00	53.00	93.50
	Mean	0.83	1.35a	2.00	25.00	70.00	5.00	40.00	90.25a
Centrale	Sonde	0.25	0.77	2.00	85.00	5.00	10.00	35	82.50
	Attibodo	0.25	0.53	1.00	65.00	25.00	10.00	40	93.6
	Wassarabou	0.25	0.49	1.00	45.00	40.00	15.00	30	88.75
	Mean	0.25	0.6b	1.33	65.00	23.33	11.66	35	88.28a
Grand Mean		0.44	0.84	1.66	38.89	51.67	9.44	51.11	81.14

**% M.C.: % mixed cropping

*Means within a column followed by the same letter(s) are not significantly different

**Fig. 4:**
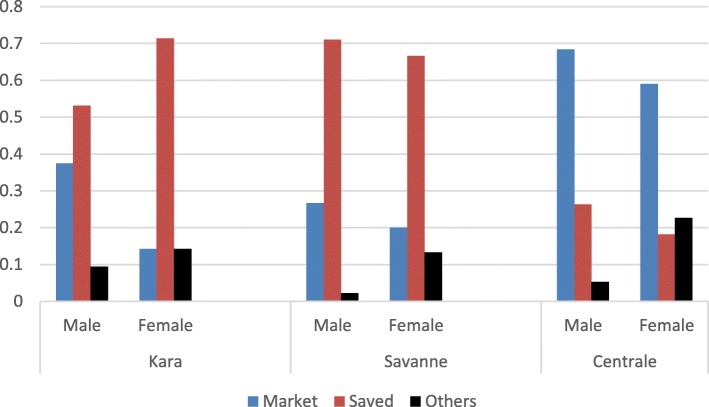
Source of groundnut seeds (in percentage of interviewees)

From the survey, 51.67% of the farmers save seeds from the previous season harvest for the next season’s farming while 38.89% buy seeds from local markets (Table 5). Only 9% of the farmers obtain seeds from other sources such as donation from Non-Governmental Organizations (NGO) and borrowing from neighboring farmers. However, at the regional level, majority of the farmers in Centrale (65%) purchase their seed from the market unlike the other regions where farmers mostly save the seeds from previous harvest (70% in Savanes and 61.66% in Kara).  Figure 4 shows a slight gender difference, that men tend to purchase seeds than women, and women tend to obtain seeds by the means of borrowing or donation than men.

**Fig. 5:**
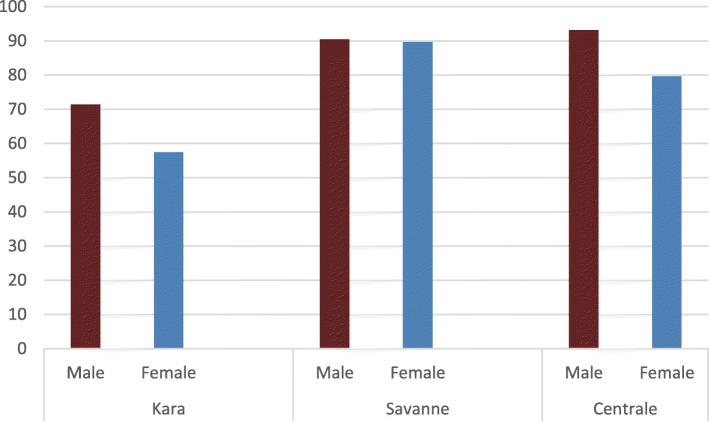
Proportion of groundnut sold (in percentage of interviewees)

Concerning cropping practice, nearly half of the farmers interviewed grow groundnut mixed with other crops such as sorghum, millet, and maize (data not shown). About 81% of the groundnut harvested in the three regions is sold at the market (Table 5). However, there is a significant difference across regions for the sold proportion of groundnut (*p* < 0.001). Groundnut is sold to a larger extent in Savanes (90%) and Centrale (88%) than in Kara (64%). Men have slightly higher rates of sold groundnut proportion in Centrale and Kara (Fig. 5).

### Cultivated groundnut varieties

A number of crops are grown by farmers in the three regions; however, maize, millet, and groundnut are the main crops. Overall, groundnut comes second in Kara and Savanes and third in Centrale. Information on the cultivated varieties in the surveyed area revealed that a small number of local groundnut landraces are grown by farmers (Table 6). In Centrale, only one variety is cultivated in each community visited. Another noticeable fact is that there is no improved variety developed and released by the national research system. Varieties such as RMP12, SORAD, and T3 were introduced a couple of years ago. SORAD, also named SOTOCO in Kara, seems to be the most widespread variety across the three regions. SORAD or SOTOCO are names of two national agricultural institutes in Togo that introduced the variety. SORAD was appreciated in the past, because of its big pod size and high yield. According to the farmers, although the pod size remained the same, the number of pods per plant has decreased resulting in a low yield.

**Table 6:** Cultivated groundnut varieties and reported characteristics by farmers

Region	Villages	Varieties	Groundnut characteristics
Savanes	Yacle	Ntifofo	
		Koka	
		Soulare	
		SORAD	Big pods and grains
	Dore	RMP12	Small pods and grains
		T3	
	Namo	SORAD	Big pods and grains
		Koumongou	
Kara	Binadjoub	SOTOCO	Big pod and grains/low yield
		Tchamba	
	Kpoloubal	Ngbengbeng	Small pods/low yield
		Djafo	
		Tchana	
	Nampoch	Smagbengbe	Small grains/low yield
		Oukandjassina	
		Tchana	
Centrale	Sonde	Nale-Nale	2 kernels per pod/low yield
	Kitambouli	Lossoketo	Small pods/low yield
	Wassarabou	SORAD	Big pod and grains

### Preferred characteristics of groundnut varieties

The analysis of the list of traits ranked by farmers shows that though there is an association between region and preferences (*χ*^*2*^(16) = 62.52, *p* < 0.001), farmers’ preferences were similar across the three regions. Generally, traits pertaining to yield are considered to be the most important. In Savanes, pod yield, pod size, and oil content were mentioned by 64.44%, 12.22%, and 6.11% of the respondents respectively (Table 7). Following pod yield (65%), drought resistance is considered to be the second most important trait in Kara. Drought (13.33%) was followed in rank by pod size and taste with 6.66% each. In Centrale, disease resistance and number of seed per pod (seed/p) were ranked third (10% each) after pod yield (61.67%) and pod size (11.66%). Other traits such as early maturity and seed color (Col.) were mentioned as secondary traits desired by the farmers. Red seed color was mostly preferred by those who mentioned color as important trait. No significant gender difference was observed for farmers’ preferred characteristics.

**Table 7:** Farmers’ preferred characteristics of groundnut varieties in percentage

Region	Village	Large pod size	Yield	High oil content	Drought	Red color	Disease resistance	Good taste	Early maturity	High no. seed/pod*
Kara	Kpoloubal	5	55	10	20	10	–	–	–	–
	Binadjoub	5	90	–	–	–	5	–	–	–
	Nampoch	10	50	–	20	–	–	20	–	–
	Mean (%)	6.66	65	3.33	13.33	3.33	1.66	6.66	–	–
Savanes	Yacle	30	60	10	–	–	–	–	–	–
	Dore	–	75	25	–	–	–	–	–	–
	Namo	25	65	10	–	–	–	–	–	–
	Mean (%)	18.33	66.66	15	–	–	–	–	–	
Centrale	Sonde	35	50	–	–	–	–	–	10	5
	Attibodo	–	70	–	–	–	5	–	10	15
	Wassarabou	–	65	–	–	–	25	–	–	10
	Mean (%)	11.66	61.67	–	–	–	10	–	6.67	10
Grand mean		12.22	64.44	6.11	4.44	1.11	3.33	2.22	2.22	3.33

*High no. seed/pod, high number of pods per plant

### Perception of farmers on groundnut production constraints

Farmers were asked to list in order of importance the main constraints of groundnut production in their areas. In most of the farmers’ opinion, diseases, insects, and drought were among the widespread constraints to groundnut production (Table 8). Indeed, diseases such as leaf spot, rosette, and peanut bud necrosis were mentioned as a major constraint on groundnut by 37.77% of the respondents. This is followed by insects (27.77%) and then drought (10.55%). Though various insects were mentioned, pod-sucking bugs (Pentatomidae; Lygaeidae) and groundnut bruchid (Bruchidae) were considered most common. Among minor insects, thrips (Thripidae) and pod borer (Noctuidae) were observed in the surveyed area. In addition, 8.33% of the interviewees pointed that the lack of high-yielding varieties is a constraint to groundnut production. Striga was ranked fifth by nearly 4.5% of the farmers as a constraint to groundnut production. There were no differences in gender.

**Table 8:** Perception of farmers on constraints to groundnut production: percentage of times that constraints were mentioned

Region	Village	Disease	Insects	Striga	Drought	Low yield	Labor	Lack of seed	Flooding	Soil fertility	No constraint
Kara	Kpoloubal	60	10	20	–	–	–	–	–	–	10
	Binadjoub	25	35	20	5	10	5	–	–	–	–
	Nampoch	30	20	–	20	20	–	–	–	–	10
	Mean	38.33	21.66	13.33	8.33	10	1.66	–	–	–	6.66
Savanne	Yacle	20	60	–	10	–	–	10	–	–	–
	Dore	25	25	–	50	–	–	–	–	–	–
	Namo	20	60	–	10	–	–	10	–	–	–
	Mean	21.66	48.33	–	23.33	–	–	6.66	–		–
Centrale	Sonde	65	15	–	–	–	–	10	10	–	–
	Attibodo	60	15	–	–	15	–	–	–	10	–
	Wassarabou	35	10	–	–	30	–	–	20	5	–
	Mean	53.33	13.33	–	–	15	–	3.33	10	5	–
Grand mean		37.77	27.77	4.44	10.55	8.33	0.55	3.33	3.33	1.66	2.22

The importance of the constraints, however, is not the same between regions (*χ*^*2*^(18) = 84.70, *p* < 0.001). In Kara and Centrale, groundnut growers ranked diseases as the major constraint. But in Savanes, diseases was ranked second by 21.66% of the interviewees behind insects (48.33%). Also, Striga was identified as one major constraint solely in Kara and ranked third by 13.33% of the farmers. The third most important constraints in Savanes and Centrale are lack of improved seed (6.66%) and high-yielding varieties (15%) respectively.

### Awareness of late leaf spot disease

Table 9 summarizes farmers’ perception of *late leaf spot (*LLS) disease. The results revealed that 95.55% of the farmers were aware of LLS disease. The awareness is the same across the three regions. However, the cause of the disease appears to be unknown to most of the farmers. Only, 39.44% of the farmers said to know the cause of the disease. And only 1.11% (Kpoloubal in Kara region) of the respondent claimed to have control measures to combat the disease.

**Table 9:** Farmers’ perception of late leaf spot (LLS)

Regions	Community	% of respondents aware of LLS symptoms	% of respondents aware the cause of LLS	% of respondents of available of control measure
Kara	Kpoloubal	100	0	10
	Binadjoub	100	10	0
	Nampoch	90	10	0
	Total	96.67	10	3.33
Savanes	Yacle	100	60	0
	Dore	100	50	0
	Namo	100	60	0
	Total	100	56.67	0
Centrale	Sonde	95	70	0
	Attibodo	85	30	0
	Wassarabou	90	55	0
	Total	90	51.67	0
Grand total		95.55	39.44	1.11

**Fig. 6:**
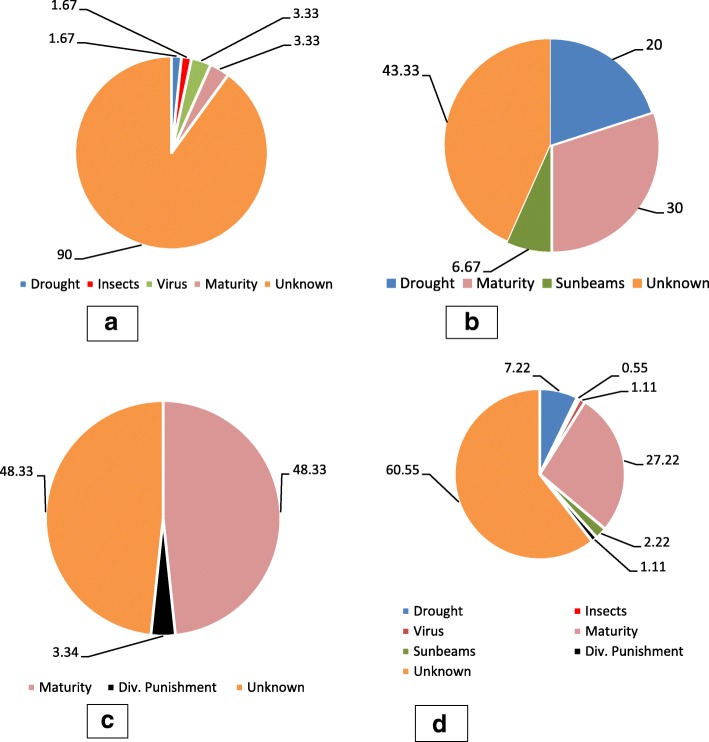
Farmers perception on the cause of LLS disease (figures are in percentage) **a** Kara; b Savanes; **c** Central; **d** Across the three regions

Between regions, however, the perception of the cause of LLS disease is not the same (*χ*^*2*^(12) = 64.04, *p* < 0.001) where 48.33% and 30% of the respondents in Centrale and Savanes respectively mentioned that LLS symptoms occurs as pod matures. But, the cause of the disease seems to be unknown by farmers in Kara (Fig.6a). In Savanes, 20% and 6.65% of the respondents associated LLS disease with drought and Sunbeams, respectively (Fig.6b). Strikingly, 3.34% of the respondents in Centrale stated divine punishment (Div. Punishment) as the probable cause of LLS disease (Fig. 6c). Across the three regions, 60.55% of the respondents did not know the cause of the disease, 27.22% of the farmers reported that the disease is associated with pod maturity while 7.22% mentioned drought to be the cause of LLS (Fig. 6d).

### Household and demographic differences, and correlations among social and farming system parameters

Household and demographic data analysis revealed significant differences in family size, age, and marital status between regions. The family size of nine in Savanes is significantly different (*p* < 0.05) from that of Centrale but similar to that of Kara. At the community level, family size in Dore is significantly higher than the other villages (*p* < 0.001) in Savanes. Age of farmers showed highly significant difference (*p* < 0.01) between regions while no significant difference was observed at the village level within region. The highest mean age was obtained in Savanes, while Kara showed the lowest mean age (results not shown). In the case of groundnut farmers’ marital status, the number of widowers in Savanes is statistically higher than those of the other regions (*p* < 0.05), and the number of single farmers in Centrale and Savanes are statistically similar but higher than that of Kara which shows the highest number of married farmers (98.33%). At the village level, there is a significant difference between communities in Savanes and Centrale on marital status. Other information collected has revealed that nearly half of the farmers are polygamous. While no statistical difference was found among regions, an association between gender and education level was observed (*χ*^*2*^(4) = 23.72, *p* < 0.001). Women were less literate than men (Fig. 2) with the highest proportion of women literacy observed in Kara.

The results also revealed that most of the farmers have been farming for more than 20 years. A significant correlation (*p* < 0.01) was observed between farmers’ age and farmers’ experience; farmers’ age and the household size, gender, and the land available for agriculture; and groundnut farm size and the land available for agriculture (Table 10).

**Table 10:** Correlation among social and farming system parameters

	Age	Household size	Sex	Land available	Education	Marital status	Groundnut land	Farmer exp
Age		***	*	**	NS	NS	***	***
Household size	0.53		NS	***	NS	***	NS	***
Sex	− 0.15	− 0.08		***	***	NS	***	NS
Land available	0.30	0.30	0.51		***	NS	***	**
Education	0.008	0.13	0.32	0.29		NS	NS	NS
Marital status	0.19	− 0.21	0.12	− 0.07	− 0.11		*	**
Groundnut land	0.19	0.04	0.36	0.63	0.12	0.15		***
Farmers’ experience	0.81	0.43	0.13	0.36	0.09	0.25	0.36	

Significance codes:***0.001; **0.01; *0.05, *NS* non-significant

## Discussion

The study not only revealed complex farming practices that show diversity and rationality but also untidy and unsystematic practices. Coupled with climate change, these farming practices may partly be responsible for the decreasing yield observed in groundnut production in Togo. New technologies for the improvement of yield are needed to quickly adapt to the changing environment. The household and demographic differences observed in this study have implications on farm size, perception of production constraints, farm productivity, and adoption of technologies. Education level affects the adoption of new technologies, and thus the strategies for introduction of new varieties and new agronomic practices should address the issue as farmers in Togo are illiterate. For example, partly due to education level, farmers in Centrale tend to purchase their seeds from markets than farmers of the other regions. These farmers are likely to adopt new varieties if there is a strong seed system. It is interesting to observe that most groundnut farmers in Centrale are native to Kara and have migrated in search for land. This is confirmed by the survey results, as Kara showed the lowest land available for agriculture. The study also revealed that land is more available to men than women showing a gender bias in accessing land for groundnut production. The significant positive correlation between land ownership and sex is an indication that gender plays an important role in land ownership. Similar findings have been reported by Katundu et al. [27], Doss et al. [28], and Kieran et al. [29]. However, in Savanes, some women own plots as large as men. Most of those women, who are financially sound, are traders living in towns who rent large plot for their groundnut production. Some respondents reported that men are abandoning groundnut production for more cashable crops such as soybean and yam because of the decreasing yield of groundnut in Nampoch. In this locality, marginalized by the development of cash crops [30], women are compelled to keep growing groundnut for home consumption justifying why there are more women in groundnut production in this area. Altogether, the current study showed a gender bias in the farmers’ educational level, access to land and groundnut seeds, confirming the necessity to develop breeding programs “with gender in mind” [31].

Farmers’ trait preferences were similar across the three regions with few exceptions. The preferred traits are pod yield, pod size, and oil content. However, in Kara, farmers considered drought resistance and taste as additional traits they want in their varieties. It is interesting to observe that farmers in Kara region consume most of the groundnut produced unlike other regions where a great proportion is sold. This could explain why taste is of great value in that region. Probably due to the market demand, the preferences of farmers in Centrale and Savanes are more diverse, as a large proportion of the harvest is sold in these regions. After yield, other traits such as pod size, disease resistance, oil content, and number of pod per plant were mentioned in these two regions. Indeed, farmers in Centrale sell their harvest in the local market for local consumption where groundnut is consumed roasted, boiled, or processed into cake. In Savanes, apart from the local consumption, a part of the production is bought by women from surrounding countries for different purposes. Throughout the surveyed area, groundnut sauce is one of the most often uses encountered. These different uses of groundnut may explain the diversity in trait preferences.

Disease, insects, and drought are the widespread constraints of groundnut production in the three regions of Togo. Similar results have been obtained during surveys in the northern regions of Ghana [32, 33], where constraints to the production were ranked as drought, yield, pests, and diseases. In Savanes, diseases were ranked second after insects in terms of importance. The agronomic practice in the Savanes may be attributed as one of the reasons why insect was ranked ahead of drought. After pulling up groundnut, farmers do not separate the pods from the plants but leave the pods attached to the haulms to dry in the field for 2 weeks. They explained that the removal of the pods from plants is easier then. At the same time, they acknowledged that pods are attacked by insects when left in the field for 1 or 2 weeks. Therefore, the identification of the insects as one of the most important constraints may be explained partly by an untidy agronomic practice. However, losses have also been associated with insect attacks at the storage level in other regions. Shortly after harvest, some farmers are, therefore, compelled to sell their groundnut at low price if they want to relieve themselves of the storage and quality maintenance problems. Other farmers are forced to sell a large proportion of their harvest soon after harvest, at low price, to honor their debts or to meet financial needs such as school fees and health care. Agricultural credits may allow farmers to boost their incomes, by keeping their harvest until the price rises. These credits are already introduced on other crops such as maize through supply of agricultural inputs and the building of storage facilities [34]. There is a need, therefore, to extend it to other crops such as groundnut. However, this will call for an integrated action including a strong research institute, an efficient extension service working regularly with farmers for coordinated agricultural activities. Though no groundnut farmers’ organization was found in the surveyed area, such an action could take advantage of the existence of other organization to which most farmers belong.

Another noticeable fact is the mention of Striga and labor as constraints of groundnut production in Kara. It is important to highlight that in Kara, more than 70% of the farmers grow groundnut in mixed cropping with sorghum. Striga is a parasitic weed that affects cereals including sorghum; therefore, the presence of Striga on their field parasitizing their sorghum plants is mistaken to also affect groundnut production. However, this offers an opportunity for the national agricultural research institute in Togo to develop a sustainable approach in addressing the problem of Striga. This also implies that sorghum varieties targeted for this region should be improved for resistance to Striga. Though, no gender difference was observed for the perception of constraint, the mention of labor as constraint in Kara could be associated to the high proportion of women in groundnut production in this area as women rely on hired people for most activities.

Late leaf spot disease, an important foliar disease of groundnut globally, was identified by farmers as the most widespread foliar disease in the three regions. This confirms other studies that have shown that late leaf spot is the most widespread foliar disease on groundnut in tropical regions [35]. Many of the farmers indicated that there is an increasing severity of the disease. According to the farmers in Centrale, about 20 years ago, there were some local resistant varieties, but these varieties are rare now as they are less adapted to the changing climate. Indeed, as most resistant landraces are late maturing varieties, the shortening of the rainy season in the surveyed area [21, 22] may explain, in part, why these varieties were abandoned and replaced by the early but more susceptible varieties. Concerning the cause of late leaf spot, the study has revealed that farmers associated the symptoms to the maturity of pods and drought. According to most of them, LLS is a natural disease which occurs when the pods are getting matured. Similar belief was reported by Izge et al. [36] in Congo on groundnut. In this study, those that associated late leaf spot to pod maturity narrated that they are oftentimes surprised to discover non-matured pods after harvest. As they tend to see late leaf spot as a natural disease, most of them overlook the effects of the disease on their crop. Other diseases such as rosette disease were mentioned by farmers and surprisingly, associated with witchcraft. These results highlight the ignorance of the farmers on the causes of LLS and other diseases. An awareness creation is required to educate farmers on these important diseases and how they can be managed for higher productivity at harvest.

The use of improved varieties was very rare as most farmers use old landraces though superior genotypes play a paramount role in achieving high productivity. The variety SOTOCO/SORAD, which was introduced into the country more than two decades ago is the most common variety found across the three regions. SOTOCO at the time of release had large pod size and high yield, and was widely adopted by farmers. Currently, farmers complained that they are no longer satisfied with the yield and expressed their desires to have new varieties that can boost their groundnut production. RMP12 and T3 are new varieties recently introduced into the country to enhance groundnut production. Unfortunately, only a few of the farmers, as the survey revealed, adopted the varieties. These varieties did not meet farmers’ preferences. It is apparent from this study that farmers in these regions would reject new varieties with small pod sizes which they associated with low yield and difficulty in manual shelling. ICIAR19B (Sumnut 24) is another variety introduced from Nigeria 4 years ago which, unfortunately, turns out to be highly susceptible to early and late leaf spot disease. These challenges offer opportunities for plant breeders in Togo to develop superior groundnut cultivars that will meet farmers’ preferences and increase the adoption rate of improved cultivars. It is important to mention here that issues such as the unavailability of groundnut seeds can be addressed through development of strong seed production systems. A breeding program that permits interaction between a range of actors including farmers, traders, and seed companies, among others, would address these issues and increase the likelihood of adoption of new groundnut varieties.

Overall, this PRA has identified the most important constraints to groundnut production in Togo. Moreover, many other limiting factors such as lack of seeds and untidy agronomic practices were revealed through this survey. Also, the perception of constraints on groundnut and of the cause of the various diseases uncovered in this study was unique in that no study has revealed such information before. Altogether, these results confirm that the PRA approach was efficient in revealing constraints to groundnut production.

## Conclusion

Information gathered from this participatory rural appraisal has revealed the farming practices, constraints in groundnut production, and farmers preferred characteristics, providing the basis for a participatory breeding program. In Togo, a breeding program on groundnut should take into account that farmers perceive diseases as the major constraints to production. In the present study, it can be inferred that high-yielding groundnut varieties with large pod size and resistance to late leaf spot are likely to be adopted by groundnut farmers in Togo.

## Abbreviations

ITRA: Institut National de Recherche Agronomique
LLS: Late leaf spot
NGO: Non-Governmental Organization
PRA: Participatory rural appraisal

## References

[1] Duraiappah AK, Roddy P, Parry JE (2005). Have participatory approaches increased capabilities? International Institute for Sustainable Development= Institut international du développement durable.

[2] Frediani AA, Boano C (2012). Processes for just products: the capability space of participatory design. The capability approach, technology and design.

[3] Abedi M, Vahidi F (2011). The importance of participatory rural appraisal (PRA) in research. J Appl Environ Biol Sci.

[4] Sattar RA, Wang S, Muqadas M, Ashraf MF, Tahir MN (2017). Qualitative and quantitative approaches to study adoption of sustainable agricultural practices: a research-note on mixed method approach. Int J Agric Ext Rural Dev.

[5] Kraaijvanger R, Veldkamp T, Almekinders C (2016). Considering change: evaluating four years of participatory experimentation with farmers in Tigray (Ethiopia) highlighting both functional and human–social aspects. Agric Syst.

[6] Kolech SA, De Jong W, Perry K, Halseth D, Mengistu F (2017). Participatory variety selection: a tool to understand farmers’ potato variety selection criteria. Open Agr.

[7] Almekinders CJ, Elings A (2001). Collaboration of farmers and breeders: participatory crop improvement in perspective. Euphytica.

[8] Morris ML, Bellon MR (2004). Participatory plant breeding research: opportunities and challenges for the international crop improvement system. Euphytica.

[9] Ortiz-Ferrara G, Bhatta MR, Pokharel T, Mudwari A, Thapa DB, Joshi AK, Chand R, Muhammad D, Duveiller E, Rajaram S (2001). Farmers’ participatory variety selection in South Asia. International maize, wheat improvement center. Research highlights of the CIMMYT wheat program 1999–2000. CIMMYT, Mexico D.F.

[10] Manzanilla DO, Paris TR, Vergara GV, Ismail AM, Pandey S, Labios RV, Tatlonghari GT, Acda RD, Chi TT, Duoangsila K, Siliphouthone I (2011). Submergence risks and farmers’ preferences: implications for breeding Sub1 rice in Southeast Asia. Agric Syst.

[11] Houngue JA, Pita JS, Cacaï GH, Zandjanakou-Tachin M, Abidjo EA, Ahanhanzo C (2018). Survey of farmers’ knowledge of cassava mosaic disease and their preferences for cassava cultivars in three agro-ecological zones in Benin. J ethnobio ethnomed.

[12] Bellon MR (2002). Analysis of the demand for crop characteristics by wealth and gender: a case study from Oaxaca, Mexico. Quantitative analysis of data from participatory methods in plant breeding.

[13] Danial D, Parlevliet J, Almekinders C, Thiele G (2007). Farmers’ participation and breeding for durable disease resistance in the Andean region. Euphytica.

[14] Aguilar A, Carranza E, Goldstein M, Kilic T, Oseni G (2015). Decomposition of gender differentials in agricultural productivity in Ethiopia. Agr Econ-Black well.

[15] Ali D, Bowen D, Deininger K, Duponchel M. Investigating the gender gap in agricultural productivity: evidence from Uganda: The World Bank; 2015.

[16] Backiny-Yetna P, McGee K. Gender differentials and agricultural productivity in Niger: The World Bank; 2015.

[17] Oseni G, Corral P, Goldstein M, Winters P. Explaining gender differentials in agricultural production in Nigeria: The World Bank; 2014. 10.1596/1813-9450-6809.

[18] Johnson NL, Kovarik C, Meinzen-Dick R, Njuki J, Quisumbing A (2016). Gender, assets, and agricultural development: lessons from eight projects. World Dev.

[19] Doss CR, Morris ML (2000). How does gender affect the adoption of agricultural innovations? The case of improved maize technology in Ghana. Agr Econ-Black-Well.

[20] Direction des Statistiques Agricoles de l’Information et de la Documentation (DSID) (2014). Principales caractéristiques de l’agriculture togolaise, 4ème recensement national de l’agriculture 2011–2014 / volume 4: module de base.

[21] Adewi E, Badameli KM, Dubreuil V (2010). Evolution of rainy seasons potentially useful in Togo from 1950 to 2000. Climatol.

[22] Ongoma V, Batebana K, Ogwang BA, Sein ZM, Ogou FK, Ngarukiyimana JP (2015). Rainfall characteristics over Togo and their related atmospheric circulation anomalies. J Environ Agr Sci.

[23] Lumley T. Complex surveys: a guide to analysis using R, vol. 565: John Wiley & Sons; 2011. https://faculty.washington.edu/tlumley/old-survey/survey-wss.pdf. Accessed 15 Jan 2018

[24] Benesty J, Chen J, Huang Y, Cohen I. Pearson correlation coefficient. In Noise reduction speech processing: Springer Berlin Heidelber; 2009. p. 1–4.

[25] Noble H, Smith J (2014). Qualitative data analysis: a practical example. Evid-Based Nurs.

[26] Wang Q (2014). Introduction to ArcGIS 10.2.

[27] Katundu MA, Mhina ML, Mbeiyererwa AG (2014). Socio-economic factors limiting smallholder groundnut production in Tabora region.

[28] Doss C, Kovarik C, Peterman A, Quisumbing A, van den Bold M (2015). Gender inequalities in ownership and control of land in Africa: myth and reality. Agric Econ.

[29] Kieran C, Sproule K, Doss C, Quisumbing A, Kim SM (2015). Examining gender inequalities in land rights indicators in Asia. Agric Econ.

[30] Wooten S. Women, men, and market gardens: gender relations and income generation in rural Mali. Hum Organ. 2003:166–77.

[31] Kristjanson P, Bryan E, Bernier Q, Twyman J, Meinzen-Dick R, Kieran C, Ringler C, Jost C, Doss C (2017). Addressing gender in agricultural research for development in the face of a changing climate: where are we and where should we be going?. Int J Agr Sust.

[32] Yussif IJ, Kwoseh C, Osman M, Acheremu K, Yirzagla J (2014). Farmers’ perception and farming practices on the effect of early and late leaf spots on groundnut production in northern Ghana. J Biol, Agr and Health.

[33] Oppong-Sekyere D, Akromah R, Akpalu MM, Ninfaa AD, Nyamah EY, Braimah MM, Salifu AR (2015). Participatory rural appraisal of constraints to groundnut (Arachis hypogaea L.) production in northern Ghana. Int J Current Res Acad Rev.

[34] ROPPA (2013). Ten years after Maputo Declaration on Agriculture and Food Security: An assessment of Progress in West Africa, Case of Togo.

[35] Sujay V, Gowda MV, Pandey MK, Bhat RS, Khedikar YP, Nadaf HL, Gautami B, Sarvamangala C, Lingaraju S, Radhakrishan T, Knapp SJ (2012). Quantitative trait locus analysis and construction of consensus genetic map for foliar disease resistance based on two recombinant inbred line populations in cultivated groundnut (Arachis hypogaea L.). Mol breeding.

[36] Izge AU, Mohammed ZH, Goni A (2007). Levels of variability in groundnut (Arachis hypogaea L.) to cercospora leaf spot disease implication for selection. Afr J Agri Res.

